# Associations of sport and exercise participation in adolescence with body composition and device-measured physical activity in adulthood: longitudinal data from the Norwegian HUNT study

**DOI:** 10.1186/s12966-025-01726-7

**Published:** 2025-03-05

**Authors:** Atle Kongsvold, Eivind Schjelderup Skarpsno, Mats Flaaten, Aleksej Logacjov, Kerstin Bach, Tom Ivar Lund Nilsen, Paul Jarle Mork

**Affiliations:** 1 Department of Public Health and Nursing, Norwegian University of Science and Technology (NTNU), Trondheim, 7491 Norway; 2 Department of Computer Science, Norwegian University of Science and Technology (NTNU), Trondheim, Norway; 3 Department of Neurology and Clinical Neurophysiology, St. Olavs Hospital, Trondheim, Norway; 4 Clinic of Emergency Medicine and Prehospital Care, St. Olavs Hospital, Trondheim, Norway

**Keywords:** Physical activity, Accelerometer, Sports participation, Body composition

## Abstract

**Background:**

To examine whether adolescent sport and exercise participation is associated with adulthood moderate-to-vigorous physical activity (MVPA), body fat, skeletal muscle mass, and body mass index (BMI), and to explore whether the association between sport and exercise participation and adult body composition depends on adulthood MVPA level.

**Methods:**

Prospective study of 4603 adolescents aged 13–18 year (57.2% female) in the Norwegian Young-HUNT Study and follow-up ~ 11 or ~ 22 years later. Linear regression was used to estimate mean differences in accelerometer-measured MVPA and bioimpedance-measured body fat, muscle mass, and BMI in adulthood according to self-reported sport and exercise participation in adolescence.

**Results:**

Adolescents participating in sport/exercise every day accumulated more MVPA (48 min/week, 95% CI 23 to 73), had less body fat (-4.4%, 95% CI -5.4 to -3.2), more muscle mass (2.6%, 95% CI 2.0 to 3.2), and lower BMI (-1.1 kg/m^2^, 95% CI -1.7 to -0.5) as adults, compared to adolescents participating < 1 day/week. Joint analysis showed that adolescents who participated in sport/exercise ≥ 4 days/week, and who accumulated 150–299 min/week MVPA in adulthood, had less body fat (-5.8%, 95% CI -7.4 to -4.3) and more muscle mass (3.4%, 95% CI, 2.5 to 4.3) compared to those participating in sport/exercise ≤ 1 day/week and who accumulated < 150 MVPA min/week as adults. Compared to the same reference group, these associations were further strengthened among those who accumulated ≥ 300 min/week MVPA in adulthood and reported ≥ 4 days/week of sport/exercise for both body fat (-8.8%, 95% CI -10.3 to -7.4) and muscle mass (5.1%, 95% CI 4.3 to 5.9).

**Conclusions:**

Adolescent sport and exercise participation is positively associated with MVPA, and skeletal muscle mass, and inversely associated with body fat and BMI in adulthood. These associations remained significant after adjusting for adult MVPA levels. A higher MVPA level in adulthood strengthens the association between adolescent sport/exercise participation and adult body composition.

**Supplementary Information:**

The online version contains supplementary material available at 10.1186/s12966-025-01726-7.

## Introduction

Despite the well-documented benefits of moderate-to-vigorous intensity physical activity (MVPA), approximately 80% of adolescents and 27% of adults fail to meet the minimum recommended MVPA level [1]. Since health behaviours established during adolescence often continue into adulthood [2, 3], promoting physical activity during these formative years can have long-term benefits, potentially lowering the risk of adverse health outcomes later in life [4].

Systematic reviews indicate that adolescents who participate in sport and exercise are more likely to maintain higher MVPA levels into young adulthood compared to their non-participating peers [2, 5]. However, most evidence relies on self-reported physical activity susceptible to measurement error [6–8]. Recent studies using device-measured physical activity show mixed results. For instance, one study found that sustained participation in youth sport predicted higher device-measured MVPA level 30 years later in females but not in males [4]. Conversely, another study found no association between sport and exercise participation at age 16 and device-measured MVPA level at age 46 [9]. Thus, the evidence appears inconclusive regarding the influence of adolescent sport and exercise participation on MVPA levels in adulthood.

In addition to the potential influence on adult physical activity level, adolescent sport and exercise participation may have additional long-term health benefits [10, 11]. For instance, a recent study found that sustained participation in sport and exercise during adolescence is inversely associated with obesity in midlife [4]. Furthermore, engaging in sport and exercise stimulates muscle growth [12] and skeletal muscle mass established during adolescence and early adulthood may be of importance for muscle strength and physical function in later adulthood [13]. However, it is currently unknown if adolescent sport and exercise participation is associated with adult muscle mass. Moreover, no study has explored whether the potential association between adolescent sport and exercise participation and adult body composition depends on adult MVPA levels.

The twofold aim of this study was to (1) examine whether adolescent sport and exercise participation is associated with adulthood MVPA, body fat, skeletal muscle mass, and body mass index (BMI), and (2) to explore whether the association between adolescent sport and exercise participation and adult body composition depends on adulthood MVPA level.

## Methods

### Study population

**Fig. 1:**
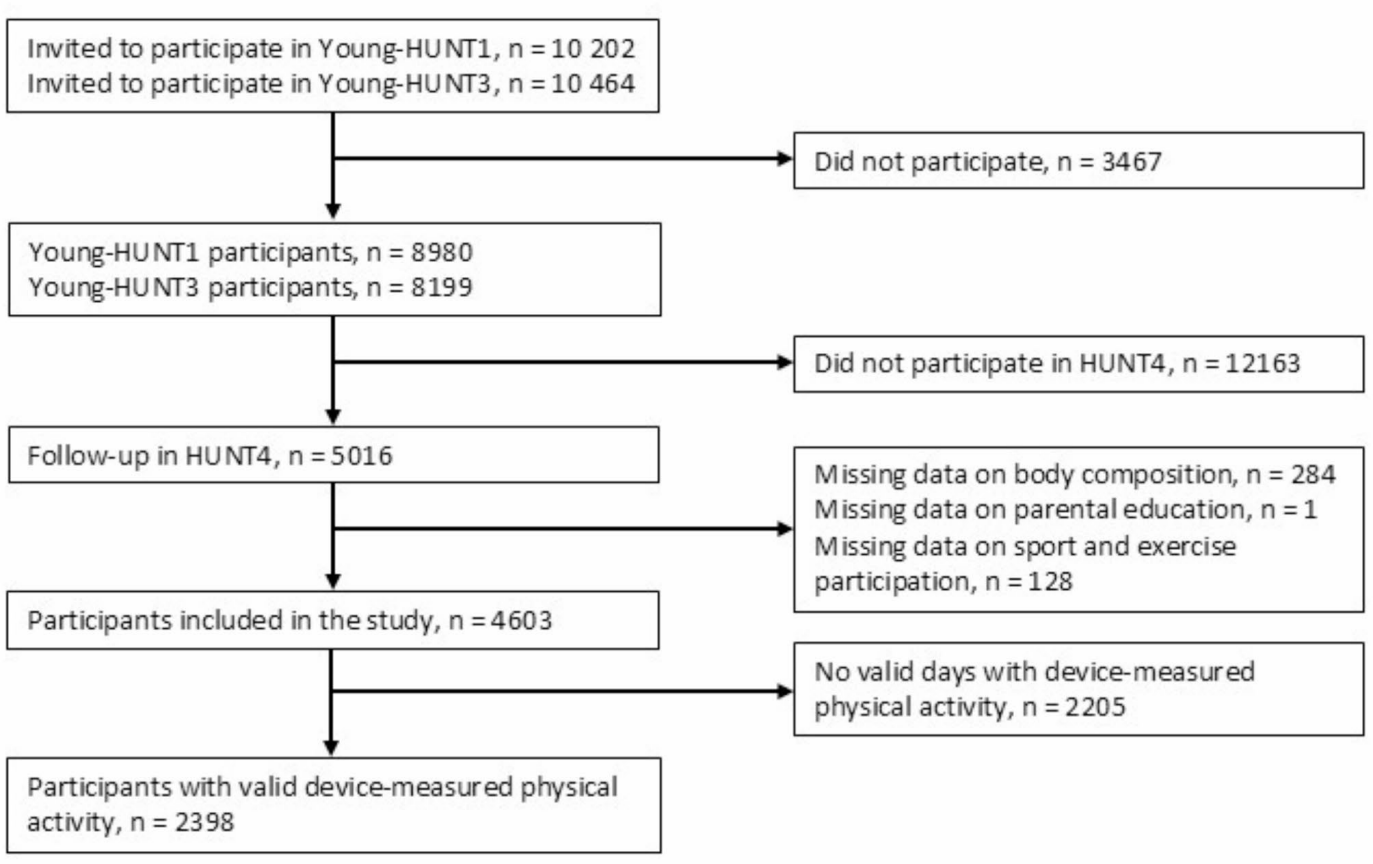
Flowchart for the selection of the study population

As part of the population-based HUNT Study, all residents aged 13–19 years in the region of Nord-Trøndelag, Norway, were invited to participate in the Young-HUNT1 (1995–1997) and Young-HUNT3 (2006–2008) surveys. Likewise, all inhabitants aged 20 years or older were invited to the HUNT4 survey (2017–2019). Detailed information about the HUNT Study can be found elsewhere [14, 15]. The flow of participants is shown in Fig. 1.

### Sport and exercise participation in adolescence

The following three questions were used to indicate sport and exercise participation at Young-HUNT1 and Young-HUNT3: (1) “How many hours per week do you play sports or exercise in your free time so much that you get out of breath or sweat?”, with response options: “<1 hour,” “1-1.5 hours,” “2–3 hours,” “4–6 hours,” or “>7 hours”, (2) “How many days per week do you play sports or exercise in your free time so much that you get out of breath or sweat?”, with response options: “<1 day,” “1 day,” “2–3 days,” “4–6 days”, or “Every day”, and (3) “Do you participate in sports competitions?”, with response options: “Yes”, “No, but I used to”, or “No”.

### Body composition in adulthood

Body fat (%), skeletal muscle mass (%), and BMI (calculated as weight divide by the square of height, kg/m^2^) were measured with bioelectrical impedance (InBody 770, Cerritos, CA, USA) at the clinical examination at HUNT4.

### Device-measured physical activity in adulthood

At HUNT4, the participants were asked to wear two triaxial AX3 accelerometers (Axivity, Ltd., Newcastle, UK) for 7 days. The procedure for collecting and processing the accelerometer data has been described elsewhere [6]. In brief, the accelerometers were attached at the lower back (third lumbar segment) and right thigh (~ 10 cm above the upper border of patella) by placing an adhesive film of 5 × 7 cm (Opsite Flexifix; Smith & Nephew, Watford, UK) directly on the skin and then covering the accelerometers with a second layer of 8 × 10 cm adhesive film. The accelerometer data was sampled at 50 Hz with 8G bandwidth. The OmGui software (version 1.0.0.37; Open Movement, Newcastle, UK) was used to configure the AX3 and download data before further processing.

The downloaded data streams were segmented into 5 s windows (250 samples) and 161 features were computed for each window. These features were fed into machine learning models trained to predict lying, sitting, standing, slow walking (< 4 km/h), moderate walking (4.1 to 5.4 km/h), brisk walking (> 5.4 km/h), running, and cycling [16–18]. Only complete days with 24 h of valid measurement were included in the analysis, i.e., the days attaching and removing the accelerometers were omitted from the analysis. Moreover, if no-wear time was predicted for ≥ 1 h, the complete 24 h period for that day was excluded from the analysis.

MVPA was defined as the sum of moderate and brisk walking, running, and cycling. We calculated MVPA min/week by dividing the number of days with valid measurement to obtain a daily average and then multiplied the daily average by seven. Participants who accumulated ≥ 150 min/week with MVPA were considered to meet current physical activity recommendations [19].

### Covariates

Information about age and sex was obtained from the HUNT Study while information on parental education was obtained by linking each participant’s record in the HUNT Study to information from Statistics Norway [20], by using the unique identification numbers held by all Norwegian citizens. Parental education was categorised as low (primary education), medium (high school), and high (university) based on the parent with highest education.

### Statistical analysis

We used linear regression to estimate mean differences in MVPA, body fat, muscle mass, and BMI according to days/week with sport and exercise participation, hours/week with sport and exercise participation, and participation in sports competition. The category indicating least sport and exercise participation was used as the reference category. To explore if the association between adolescent sport and exercise participation and body composition in adulthood depends on MVPA levels in adulthood, we first categorized the adolescent sport and exercise participation into *≤* 1 day/week, 2–3 days/week, and *≥* 4 days/week, and thereafter combined these categories with device-measured MVPA levels in adulthood, i.e., < 150 min/week, 150–299 min/week, and *≥* 300 min/week. In these analyses, participants who reported *≤* 1 day/week of adolescent sport and exercise participation and had < 150 MVPA min/week as an adult was used as the reference category. We used a likelihood ratio test that includes the product term between two variables to evaluate the possible interaction of sport and exercise participation in adolescence and device-measured MVPA level in adulthood with body fat, skeletal muscle mass, and BMI in adulthood. All association were adjusted for age at baseline (years), sex (male, female), baseline HUNT survey (Young-HUNT1, Young-HUNT3), and parental education (low, medium, high). In supplementary analysis, we stratified according to baseline HUNT survey to assess whether the difference in follow-up time influenced the results. In analysis where body composition (i.e., body fat, muscle mass, BMI) was the outcome, we conducted an additional analysis adjusting for the adult MVPA level. Additionally, we adjusted for baseline BMI (continuous) in sensitivity analysis. To confirm that the underlying assumptions for linear regression were met, all variables were examined for normality of residuals and heterogeneity of variance. In additional analysis, we calculated the transition probabilities using the proportion command in Stata for four trajectories; active in adolescence and active in adulthood, active in adolescence and inactive in adulthood, inactive in adolescence and active in adulthood, and inactive in adolescence and inactive in adulthood. We defined ≥ 4 days/week of sport/exercise participation as being active in adolescence and ≥ 150 min/week MVPA as being active in adulthood. All statistical analysis was performed using Stata for Windows, version 18.0/MP (StataCorp LP, College Station, Texas, USA).

## Results

Table 1 shows the characteristics of the study population, stratified by baseline survey and days/week of adolescent sport and exercise participation. A total of 4603 people (57.3% female) with a mean baseline age of 16.1 (SD 1.8) years were included in the study. The mean age at follow-up for those who participated in Young-HUNT1 was 37.9 (SD 1.9) years and 26.7 (SD 1.9) years for those who participated in Young-HUNT3. The mean number of valid days with accelerometer recordings was 5.4 (SD 1.3, range 1–7). Characteristics of the study population, stratified by hours/week of adolescent sport and exercise participation and participation in sport competitions is presented in Supplementary Table 1.

**Table 1:** Characteristics of the study population stratified by baseline survey and days per week of adolescent sport and exercise participation

Characteristic	All	Young-HUNT1	Young-HUNT3	Sport and exercise participation (days/week)				
				< 1 day	1 day	2–3 days	4–6 days	Every day
No. (%)	4603 (100)	2594 (56.4)	2009 (43.7)	673 (14.6)	645 (14.0)	1790 (38.9)	1138 (24.7)	357 (7.8)
Sex								
Male, no. (%)	1968 (42.8)	1119 (56.0)	849 (44.0)	289 (14.7)	247 (12.6)	705 (35.8)	512 (26.0)	215 (10.9)
Female, no. (%)	2635 (57.3)	1475 (56.9)	1160 (43.1)	384 (14.6)	398 (15.1)	1085 (41.2)	626 (23.8)	142 (5.4)
Age								
At baseline, mean (SD), years	16.1 (1.8)	16.2 (1.8)	15.9 (1.8)	16.6 (1.8)	16.4 (1.8)	15.9 (1.8)	15.9 (1.8)	16.0 (1.8)
At follow-up, mean (SD), years	33.0 (5.9)	37.9 (1.9)	26.7 (1.9)	34.2 (5.7)	33.9 (5.7)	33.3 (5.7)	32.0 (6.0)	31.1 (6.0)
Body mass index								
At baseline^*^, mean (SD), kg/m^2^	21.6 (3.3)	21.3 (3.0)	22.1 (3.5)	21.8 (3.8)	22.2 (3.6)	21.7 (3.6)	21.6 (3.1)	21.4 (2.8)
At follow up, mean (SD), kg/m^2^	26.1 (4.8)	26.6 (4.7)	25.6 (4.9)	27.1 (5.2)	26.9 (5.2)	26.3 (5.1)	25.8 (4.5)	25.7 (4.9)
Parental educational level								
Primary school, no. (%)	297 (6.5)	194 (65.3)	103 (34.7)	60 (20.2)	43 (14.5)	119 (40.1)	53 (17.9)	22 (7.4)
High school, no. (%)	2644 (57.4)	1589 (60.1)	1055 (39.9)	422 (16.0)	400 (15.1)	1056 (40.0)	595 (22.5)	171 (6.5)
University, no. (%)	1662 (36.0)	811 (48.8)	851 (51.2)	191 (11.5)	202 (12.2)	615 (37.0)	490 (29.5)	164 (9.9)

^*^ 229 people with missing data

SD, standard deviation

Adolescent sport and exercise participation was positively associated with adulthood MVPA level (Table 2). Compared to adolescents who participated < 1 day/week, those who participated 2–3 days/week, 4–6 days/week, or every day accumulated 23 min (95% CI, 6 to 40), 47 min (95% CI, 29 to 65), and 48 min (95% CI, 23 to 73) more MVPA/week as adults, respectively. Hours/week with sport and exercise participation and participation in sport competitions were also positively associated with MVPA (Table 2). The difference in age at follow-up (i.e., ~ 38 vs. ~27 years) had minor influence on these associations (Supplementary Table 2). Likewise, there was no discernible gender difference for these associations (Supplementary Table 3). Additionally, results remained essentially unchanged after adjusting for baseline BMI, e.g., mean difference increased to 51 MVPA min/week (95% CI, 25 to 76) for those who participated daily in sport and exercise. The proportion of participants that was active both in adolescence and adulthood was 28%, while only 4% who were active in adolescence became inactive as an adult (Supplementary Table 4). Correspondingly, 14% were inactive both as an adolescent and adult while 54% became active as an adult.

**Table 2:** The association between adolescent sport and exercise participation and minutes per week with device-measured moderate-to-vigorous physical activity in adulthood

Sport and exercise participation	No. of people	Mean MVPA min/week	Crude mean difference	Adjusted* mean difference (95% CI)
Days per week				
<1 day	366	222	0.0 (ref.)	0.0 (ref.)
1 day	319	224	2	3 (-18 to 24)
2–3 days	938	244	22	23 (6 to 40)
4–6 days	597	270	48	47 (29 to 65)
Every day	178	274	52	48 (23 to 73)
Hours per week				
<1 h	381	227	0.0 (ref.)	0.0 (ref.)
1–1.5 h	346	226	-1	1 (-19 to 21)
2–3 h	624	241	14	14 (-4 to 32)
4–6 h	660	251	24	25 (8 to 43)
≥7 h	390	288	61	57 (38 to 77)
Competing in sports^†^				
No	308	220	0.0 (ref.)	0.0 (ref.)
No, but used to	782	235	16	11 (-7 to 30)
Yes	1124	264	44	40 (22 to 58)

^*^ Adjusted for baseline age (continuous), sex (male, female), HUNT survey (Young-HUNT1, Young-HUNT3), and parental education (low, medium, high)

^†^ 184 people with missing data

CI, Confidence interval; MVPA, moderate-to-vigorous intensity physical activity

Adolescents who participated in sport and exercise 2–3 days/week had 1.9% (95% CI, -2.7 to -1.2) less body fat, 1.2% (95% CI, 0.8 to 1.6) more muscle mass, and a 0.6 kg/m^2^ (95% CI, -1.0 to -0.1) lower BMI as adults, compared to those who participated < 1 day/week (Table 3). For those who participated every day, these differences increased to 4.4% (95% CI, -5.4 to -3.2) less body fat, 2.6% (95% CI, 2.0 to 3.2) more muscle mass, and a 1.1 kg/m^2^ (95% CI, -1.7 to -0.5) lower BMI. Adjusting for adult MVPA led to somewhat weaker associations, e.g., compared to those participating < 1 day/week, those participating every day had 3.6% (95% CI, -5.6 to -2.7) less body fat, 2.2% (95% CI, 1.4 to 3.0) more muscle mass and 0.5 kg/m^2^ (95% CI, -1.4 to 0.4) lower BMI. Similar associations were observed for hours/week with sport and exercise participation and for participation in sport competitions during adolescence. Additional adjustment for baseline BMI did not alter these associations, i.e., among those who participated daily in sport and exercise, the mean differences were 4.4% (95% CI -5.4 to -3.2) less body fat, 2.6% (95% CI 2.0 to 3.2) more muscle mass, and 1.1 kg/m^2^ (-1.7 to -0.5) lower BMI. However, all associations were attenutated with longer follow-up time, i.e., mean differences were less pronounced for participants in Young-HUNT1 compared to Young-HUNT3 (Supplementary Tables 5–7). There were no discernable systematic gender differences for the association between adolescent sport and exercise participation and adulthood body composition (Supplementary Tables 8 and 9).

**Table 3:** The association between adolescent sport and exercise participation and body fat, skeletal muscle mass, and body mass index in adulthood

Sports and exercise participation	No. of people	Mean body fat (%)	Crude mean difference	Adjusted^*^ mean difference (95% CI)		Mean muscle mass (%)	Crude mean difference	Adjusted^*^ mean difference (95% CI)		Mean BMI (kg/m^2^)	Crude mean difference	Adjusted^*^ mean difference (95% CI)
Days per week												
<1 day	673	30.4	0.0 (ref.)	0.0 (ref.)		38.8	0.0 (ref.)	0.0 (ref.)		27.1	0.0 (ref.)	0.0 (ref.)
1 day	645	30.2	-0.2	-0.6 (-1.5 to 0.3)		38.9	0.1	0.4 (-0.1 to 0.9)		26.9	-0.2	-0.1 (-0.6 to 0.4)
2–3 days	1790	28.6	-1.8	-1.9 (-2.7 to -1.2)		39.8	1.0	1.2 (0.8 to 1.6)		26.3	-0.8	-0.6 (-1.0 to -0.1)
4–6 days	1138	26.0	-4.3	-3.6 (-4.4 to -2.8)		41.4	2.6	2.2 (1.7 to 2.6)		25.8	-1.3	-1.0 (-1.4 to -0.5)
Every day	357	23.7	-6.7	-4.4 (-5.4 to -3.2)		42.9	4.1	2.6 (2.0 to 3.2)		25.7	-1.4	-1.1 (-1.7 to -0.5)
Hours per week												
<1 h	748	30.2	0.0 (ref.)	0.0 (ref.)		38.9	0.0 (ref.)	0.0 (ref.)		27.2	0.0 (ref.)	0.0 (ref.)
1–1.5 h	660	30.1	-0.1	-0.7 (-1.6 to 0.2)		38.9	0.0	0.4 (-0.1 to 0.9)		26.6	-0.6	-0.3 (-0.8 to 0.3)
2–3 h	1200	29.0	-1.2	-1.5 (-2.3 to -0.8)		39.6	0.7	0.9 (0.5 to 1.4)		26.4	-0.8	-0.6 (-1.0 to -0.1)
4–6 h	1243	27.0	-3.1	-3.1 (-3.8 to -2.3)		40.7	1.8	1.8 (1.4 to 2.2)		25.9	-1.3	-1.0 (-1.4 to -0.5)
≥7 h	752	24.5	-5.7	-4.0 (-4.9 to -3.2)		42.4	3.5	2.4 (2.0 to 2.9)		25.9	-1.3	-1.0 (-1.5 to -0.5)
Competing in sports^†^												
No	601	30.6	0.0 (ref.)	0.0 (ref.)		38.6	0.0 (ref.)	0.0 (ref.)		26.7	0.0 (ref.)	0.0 (ref.)
No, but used to	1496	29.2	-1.4	-0.9 (-1.7 to -0.1)		39.5	0.9	0.6 (0.1 to 1.0)		26.6	-0.1	-0.2 (-0.7 to 0.2)
Yes	2106	26.4	-4.3	-3.3 (-4.0 to -2.5)		41.2	2.6	1.9 (1.5 to 2.4)		25.8	-0.9	-1.0 (-0.7 to -0.5)

^*^ Adjusted for baseline age (continuous), sex (male, female), HUNT survey (Young-HUNT1, Young-HUNT3), and parental education (low, medium, high)

^†^ 400 people with missing data

CI, Confidence interval; BMI, body mass index

Table 4 shows the association between adolescent sport and exercise participation and adulthood body composition according to MVPA level in adulthood. Although there was no evidence of statistical interaction when including those who participated *≤* 1 day/week versus *≥* 4 days/week in adolescense, and who accumulated *≤* 150 min/week versus *≥* 300 min/week MVPA in adulthood, we obsereved that those who participated ≥ 4 days/week and accumulated ≥ 300 MVPA min/week, had 8.8% (95% CI -10.3 to -7.4) less body fat, compared to the reference group who particpated ≤ 1 day/week and accumulated < 150 MVPA min/week. The corresponding difference for those who participated ≤ 1 day/week and with the same MVPA level as an adult, was 5.0% (95% CI -6.6 to -3.4) less body fat. Similar patterns were observed for muscle mass and BMI, i.e., those who participated ≥ 4 days/week tended to have more muscle mass and lower BMI in adulthood compared to those who participated ≤ 1 day/week and with the same adult MVPA level.

**Table 4:** The joint association between adolescent sport and exercise participation and device-measured moderate-to-vigorous physical activity in adulthood, with body fat, skeletal muscle mass, and body mass index in adulthood

Sport and exercise participation and MVPA min/week	No. of people	Mean body fat (%)	Crude mean difference	Adjusted^*^ mean difference (95% CI)		Mean muscle mass (%)	Crude mean difference	Adjusted^*^ mean difference (95% CI)		Mean BMI (kg/m^2^)	Crude mean difference	Adjusted^*^ mean difference (95% CI)
*≤* 1 day per week												
<150 min	163	34.3	0.0 (ref.)	0.0 (ref.)		36.4	0.0 (ref.)	0.0 (ref.)		28.2	0.0 (ref.)	0.0 (ref.)
150–299 min	290	29.4	-4.9	-4.4 (-6.0 to -2.9)		39.2	2.8	2.4 (1.6 to 3.3)		25.8	-2.5	-2.5 (-3.4 to -1.6)
*≥*300 min	232	28.1	-6.2	-5.0 (-6.6 to -3.4)		40.1	3.7	2.8 (1.9 to 3.7)		26.4	-1.8	-2.0 (-2.9 to -1.0)
2–3 days per week												
<150 min	160	33.2	-1.1	-1.4 (-3.2 to 0.3)		37.1	0.7	0.9 (-0.1 to 1.8)		27.5	-0.7	-0.6 (-1.6 to 0.4)
150–299 min	383	29.8	-4.5	-4.2 (-5.6 to -2.7)		39.0	2.6	2.4 (1.6 to 3.2)		26.4	-1.9	-1.8 (-2.7 to -1.0)
*≥*300 min	395	25.6	-8.7	-7.2 (-8.6 to -5.7)		41.5	5.1	4.1 (3.3 to 4.9)		25.3	-2.9	-2.9 (-3.8 to -2.1)
*≥* 4 days per week												
<150 min	106	30.7	-3.6	-3.4 (-5.4 to -1.5)		38.5	2.0	2.0 (0.9 to 3.0)		26.5	-1.7	-1.5 (-2.6 to -0.3)
<150–299 min	281	26.9	-7.4	-5.8 (-7.4 to -4.3)		40.9	4.4	3.4 (2.5 to 4.2)		26.0	-2.2	-2.1 (-3.1 to -1.2)
≥300 min	388	22.9	-11.5	-8.8 (-10.3 to -7.4)		43.3	6.9	5.1 (4.3 to 5.9)		25.1	-3.1	-3.0 (-3.9 to -2.2)

^*^ Adjusted for baseline age (continuous), sex (male, female), HUNT survey (Young-HUNT1, Young-HUNT3), and parental education (low, medium, high)

CI, Confidence interval; MVPA, moderate-to-vigorous intensity physical activity

## Discussion

We found strong associations between adolescent sport and exercise participation and indicators of adult physical health. Adolescents who engaged daily in sport and exercise had higher device-measured MVPA level (mean difference 48 min/week), lower body fat (mean difference 4.4%), more skeletal muscle mass (mean difference 2.6%), and lower BMI (mean difference 1.1 kg/m^2^) in adulthood, compared to those who participated < 1 day/week. The association between adolescent sport and exercise participation and adult body composition was to some extent dependent on adult MVPA level, i.e., the increments in a favourable body composition were somewhat more pronounced among those with high versus low sport and exercise participation given the same increase in adulthood MVPA levels.

Our results expand on previous research indicating that early sport and exercise participation can have lasting positive effects on adult physical activity level [5]. Compared to previous studies, the questions used in the HUNT Study, along with the large study sample, allowed for a more comprehensive analysis of the association between adolescent sport and exercise participation and adult MVPA level. While we used information about number of days (< 1 day to 7 days) and hours (< 1 h to ≥ 7 h) per week, previous studies have typically used a narrower scale to indicate adolescent sport and exercise participation. For example, in a recent study based on the 1970 British Cohort Study, the category indicating the highest frequency of participation was “More than once a week” [9]. This may in part explain why they did not observe an association between sport and exercise participation at age 16 years and MVPA level at age 46 years. Additionally, the difference in follow-up time (i.e., 30 years vs. ~ 17 years in the current study) may also account for the contrasting findings. However, it should be noted that we did not find a weaker association between adolescent sport and exercise participation and MVPA level at age ~ 38 years compared to at age ~ 27 years. Although we cannot rule out a birth cohort effect on adulthood MVPA level (i.e., an overall decline in MVPA in Young-HUNT3 participants compared to Young-HUNT1 participants) as shown by other studies for the same time period [21], our findings suggest that adolescent sport and exercise participation strongly predict adulthood MVPA level at least until the late thirties. The long-lasting benefit on adult physical activity level is supported by findings in the Young Finns Study, showing that sustained sport participation in youth was associated with higher device-measured MVPA level among females at age ~ 48 years [4, 22]. 

Some studies link adolescent sport and exercise participation to a favourable adult body composition [10, 11], while others have reported no association [23]. This discrepancy may be due to methodological differences, e.g., the study reporting no association assessed sport engagement by summing up the number of sports the adolescents were taking part in [23]. In comparison, we assessed adolescent participation by weekly frequency (days), weekly duration (hours), and participation in competitions. Overall, both weekly frequency and duration were dose-dependently associated with body fat, skeletal muscle mass, and BMI in adulthood. Although the associations were strongest for those who participated daily or ≥ 7 h/week, those who participated 2–3 days or 2–3 h per week also had significantly lower body fat, more muscle mass, and lower BMI in adulthood compared to those with least participation. In addition, adjusting for adult MVPA levels had modest impact on the associations, indicating that sport and exercise participation during adolescence may have a direct effect on adulthood body composition, and not only through adulthood MVPA.

Our joint analysis of the association between adolescent sport and exercise participation and adult MVPA level on adult body composition indicates that a high adulthood MVPA level strengthens these associations. Although our study suggests that remaining highly active during adolescence and into adulthood conferred the largest benefits on adult body composition, we also observed that becoming active in adulthood has significant benefits. For instance, those who were active *≤* 1 day/week during adolescence but met the MVPA recommendations in adulthood had a more favourable body composition than those who remained inactive into adulthood. Thus, although a lifelong active lifestyle is most beneficial it is important to note that becoming active as an adult have substantial health benefits. Furthermore, in the analysis stratified by HUNT survey, we observed that the associations between adolescent sport and exercise participation and adult body composition were attenuated with extended follow-up time. Conversely, when stratifying the joint analysis by HUNT survey, these associations became somewhat stronger for the longer follow-up, indicating that adolescent sport and exercise participation combined with high adulthood MVPA may enhance the long-term benefits on body composition.

Some limitations should be considered when interpreting the results. First, adolescent sport and exercise participation was measured only once, and we cannot determine whether the duration of participation influences the association with adulthood MVPA and body composition. Second, the measurements of adulthood MVPA and body composition were conducted at the same time point. It is possible that having an unfavourable body composition is a cause of a lower adult physical activity level, thereby leading to an overestimation of the influence of MVPA on body composition. Third, we have no information about the type of sport or exercise the adolescents were engaged in. Some evidence suggests that the type of sport or exercise influences the association with adult physical activity level [24]. Fourth, although our data allowed for follow-up until the late thirties, we cannot conclude whether the observed associations are sustained into middle age and older adulthood. Fifth, we cannot rule out residual confounding due to unknown or unmeasured factors. For example, an active lifestyle during adolescence can also be associated with a healthier diet that is carried on into adulthood, thereby influencing adult body composition. These associations can also be distorted by genetic liability to physical activity or unfavourable body composition. Finally, we cannot rule out that the exclusion of participants due to missing data or the drop-out during follow-up biased our estimates. However, such bias would require that the exclusion and/or drop-out is differential between the categories of sport and exercise participation. For instance, if those who were highly active during adolescence but inactive in adulthood are overrepresented among those who dropped out, this would overestimate the positive association between adolescent sport and exercise participation and the adult MVPA level.

This study highlights the potential long-term benefits of sport and exercise during adolescence on body composition and physical activity level in adulthood. The findings underscore the importance of measures that promote and reinforce sport and exercise participation in adolescents to enhance public health. Notably, a high physical activity level in adulthood seems to strengthen the positive association between adolescent sport and exercise participation and a favourable body composition in adulthood. Further research aimed at developing and testing interventions that promote and reinforce sustained physical activity from adolescence into adulthood could provide valuable insights for public health strategies. Although we observed strong associations between adolescent sport and exercise participation and adult MVPA levels and body composition, we also found that 54% of those defined as being inactive in adolescence (i.e., ≤ 4 days/week sport and exercise participation) fulfilled the physical activity recommendations as an adult. This suggests that interventions targeting adults who were previously inactive to become active are highly feasible. Notably, only 4% of those who were active as adolescents became inactive as adults, underscoring the potential importance of promoting sport and exercise participation in childhood and adolescence.

## Conclusion

This prospective cohort study demonstrates strong dose-dependent associations between adolescent sport and exercise participation and adult MVPA level, body fat, skeletal muscle mass, and BMI. These associations remained significant after adjusting for adult MVPA levels. Higher levels of adulthood MVPA may contribute to strengthen the association between adolescent sport and exercise participation and adult body composition.

## Electronic supplementary material

Below is the link to the electronic supplementary material.

Supplementary Material 1

## Data Availability

No datasets were generated or analysed during the current study.

## Abbreviations

BMI: Body mass index
CI: Confidence interval
HUNT: The Trøndelag Health Study
MVPA: Moderate-to-vigorous intensity physical activity
SD: Standard deviation
