# Supplementary material for: Associations of sport and exercise participation in adolescence with body composition and device-measured physical activity in adulthood: longitudinal data from the Norwegian HUNT study
Source: Int J Behav Nutr Phys Act. 2025 Mar 5;22:29. doi: 10.1186/s12966-025-01726-7 (PMC11883909; doi:10.1186/s12966-025-01726-7)
Supplement: Supplementary file 1 — Supplementary Material 1 [file 12966_2025_1726_MOESM1_ESM.docx]

Supplementary Table 1. Characteristics of the study population stratified by hours per week of adolescent sport and exercise participation and participation in sports competitions.

| Characteristic | Sport and exercise participation (hours/week) | | | | |  | Participating in competitions^†^ | | |
| --- | --- | --- | --- | --- | --- | --- | --- | --- | --- |
|  | <1 hr | 1-1.5 hrs | 2-3 hrs | 4-6 hrs | ≥7 hrs |  | Yes | No, but used to | No |
| No. (%) | 748 (16.3) | 660 (14.3) | 1200 (26.1) | 1243 (27.0) | 752 (16.3) |  | 2106 (50.1) | 1496 (35.6) | 601 (14.3) |
| Sex |  |  |  |  |  |  |  |  |  |
| Male, no. (%) | 336 (17.1) | 235 (11.9) | 469 (23.8) | 517 (26.3) | 411 (20.9) |  | 943 (53.1) | 620 (34.9) | 212 (11.9) |
| Female, no. (%) | 412 (15.6) | 425 (16.1) | 731 (27.7) | 726 (27.6) | 341 (12.9) |  | 1163 (53.3) | 876 (36.1) | 389 (16.0) |
| Age |  |  |  |  |  |  |  |  |  |
| At baseline, mean (SD), years | 16.4 (1.9) | 16.1 (1.9) | 16.1 (1.8) | 15.9 (1.7) | 16.2 (1.8) |  | 15.7 (1.7) | 16.6 (1.8) | 16.0 (1.8) |
| At follow-up, mean (SD), years | 34.3 (5.7) | 32.6 (5.8) | 33.8 (5.7) | 32.7 (5.8) | 31.6 (6.0) |  | 32.5 (4.9) | 33.4 (5.9) | 31.4 (5.6) |
| Body mass index |  |  |  |  |  |  |  |  |  |
| At baseline^*^, mean (SD), kg/m^2^ | 21.9 (4.0) | 22.0 (3.7) | 21.7 (3.5) | 21.5 (3.1) | 21.8 (3.1) |  | 21.3 (3.0) | 22.2 (3.6) | 22.3 (4.1) |
| At follow up, mean (SD), kg/m^2^ | 27.2 (5.5) | 26.6 (5.5) | 26.4 (4.9) | 25.9 (4.7) | 25.9 (4.5) |  | 25.8 (4.6) | 26.7 (5.2) | 26.7 (5.6) |
| Parental educational level |  |  |  |  |  |  |  |  |  |
| Primary school, no. (%) | 60 (9.9) | 38 (5.8) | 75 (6.3) | 80 (6.4) | 30 (4.0) |  | 108 (41.5) | 108 (41.5) | 44 (16.9) |
| High school, no. (%) | 474 (63.4) | 399 (60.5) | 707 (58.9) | 690 (55.5) | 374 (49.7) |  | 1141 (48.0) | 874 (36.7) | 364 (15.3) |
| University, no. (%) | 200 (26.7) | 223 (33.8) | 418 (34.8) | 473 (38.1) | 348 (46.3) |  | 857 (54.8) | 514 (32.9) | 193 (12.3) |

^*^ 229 people with missing data.

^†^ 400 people with missing data

SD, standard deviation

Supplementary Table 2. The association between adolescent sport and exercise participation and device-measured moderate-to-vigorous physical activity (MVPA) in adulthood, stratified by baseline HUNT survey.

|  | Young HUNT1 | | | |  | Young HUNT3 | | | |
| --- | --- | --- | --- | --- | --- | --- | --- | --- | --- |
| Sport and exercise participation | No. of people | Mean  MVPA min/week | Crude mean difference | Adjusted^*^ mean difference (95% CI) |  | No. of people | Mean  MVPA min/week | Crude mean difference | Adjusted^*^ mean difference (95% CI) |
| Days per week |  |  |  |  |  |  |  |  |  |
| <1 day | 228 | 226 | 0.0 (ref.) | 0.0 (ref.) |  | 138 | 215 | 0.0 (ref.) | 0.0 (ref.) |
| 1 day | 199 | 225 | -1 | 1 (-25 to 27) |  | 120 | 222 | 7 | 6 (-29 to 41) |
| 2-3 days | 594 | 246 | 20 | 23 (2 to 44) |  | 344 | 241 | 26 | 24 (-4 to 53) |
| 4-6 days | 297 | 278 | 52 | 50 (27 to 74) |  | 300 | 263 | 48 | 43 (14 to 72) |
| Every day | 75 | 270 | 44 | 41 (6 to 76) |  | 103 | 277 | 63 | 50 (13 to 88) |
| Hours per week |  |  |  |  |  |  |  |  |  |
| <1 hr | 252 | 232 | 0.0 (ref.) | 0.0 (ref.) |  | 129 | 217 | 0.0 (ref.) | 0.0 (ref.) |
| 1-1.5 hrs | 183 | 218 | -14 | -12 (-37 to 15) |  | 163 | 235 | 17 | 16 (-18 to 49) |
| 2-3 hrs | 401 | 248 | 16 | 18 (-3 to 39) |  | 223 | 228 | 10 | 8 (-24 to 39) |
| 4-6 hrs | 382 | 252 | 21 | 22 (0 to 43) |  | 278 | 250 | 33 | 31 (1 to 61) |
| ≥7 hrs | 175 | 295 | 63 | 60 (34 to 86) |  | 212 | 282 | 65 | 55 (24 to 87) |
| Competing in sports^†^ | |  |  |  |  |  |  |  |  |
| No | 139 | 204 | 0.0 (ref.) | 0.0 (ref.) |  | 169 | 233 | 0.0 (ref.) | 0.0 (ref.) |
| No, but used to | 439 | 242 | 39 | 31 (5 to 57) |  | 343 | 264 | -7 | -8 (-34 to 19) |
| Yes | 645 | 264 | 60 | 55 (30 to 80) |  | 479 | 264 | 31 | 25 (-1 to 51) |

^*^ Adjusted for baseline age (continuous), sex (male, female), HUNT survey (Young-HUNT1, Young-HUNT3), and parental education (low, medium, high)

^†^ 184 people with missing data

Supplementary Table 3. The association between adolescent sport and exercise participation and device-measured moderate-to-vigorous physical activity (MVPA) in adulthood, stratified by gender.

|  | Females | | | |  | Males | | | |
| --- | --- | --- | --- | --- | --- | --- | --- | --- | --- |
| Sport and exercise participation | No. of people | Mean  MVPA min/week | Crude mean difference | Adjusted^*^ mean difference (95% CI) |  | No. of people | Mean  MVPA min/week | Crude mean difference | Adjusted^*^ mean difference (95% CI) |
| Days per week |  |  |  |  |  |  |  |  |  |
| <1 day | 223 | 207 | 0.0 (ref.) | 0.0 (ref.) |  | 143 | 245 | 0.0 (ref.) | 0.0 (ref.) |
| 1 day | 215 | 221 | 13 | 12 (-13 to 38) |  | 104 | 231 | -14 | -15 (-51 to 21) |
| 2-3 days | 601 | 234 | 27 | 28 (7 to 49) |  | 337 | 262 | 17 | 16 (-12 to 45) |
| 4-6 days | 339 | 257 | 50 | 51 (28 to 74) |  | 258 | 287 | 42 | 42 (12 to 72) |
| Every day | 78 | 231 | 24 | 24 (-11 to 59) |  | 100 | 307 | 62 | 64 (27 to 102) |
| Hours per week |  |  |  |  |  |  |  |  |  |
| <1 hr | 227 | 215 | 0.0 (ref.) | 0.0 (ref.) |  | 154 | 244 | 0.0 (ref.) | 0.0 (ref.) |
| 1-1.5 hrs | 235 | 223 | 8 | 7 (-18 to 32) |  | 111 | 231 | -13 | -13 (-48 to 22) |
| 2-3 hrs | 397 | 226 | 11 | 10 (-12 to 33) |  | 227 | 267 | 23 | 21 (-8 to 51) |
| 4-6 hrs | 408 | 242 | 27 | 28 (6 to 50) |  | 252 | 267 | 23 | 22 (-7 to 51) |
| ≥7 hrs | 189 | 266 | 51 | 50 (23 to 76) |  | 198 | 310 | 66 | 66 (36 to 96) |
| Competing in sports^†^ | |  |  |  |  |  |  |  |  |
| No | 210 | 216 | 0.0 (ref.) | 0.0 (ref.) |  | 98 | 227 | 0.0 (ref.) | 0.0 (ref.) |
| No, but used to | 479 | 223 | 6 | 3 (-19 to 26) |  | 303 | 255 | 28 | 28 (-5 to 61) |
| Yes | 657 | 248 | 31 | 31 (9 to 52) |  | 467 | 287 | 59 | 59 (28 to 91) |

^*^ Adjusted for baseline age (continuous), HUNT survey (Young-HUNT1, Young-HUNT3), and parental education (low, medium, high)

^†^184 people with missing data

Supplementary Table 4. Transition probabilities between adolescent sport and exercise participation and adulthood MVPA level.

| Sport and exercise participation as adolescent | MVPA level as adult | Transition probabilities (95% CI) |
| --- | --- | --- |
| Active (≥4 days per week) | Active (≥150 min/week) | 0.28 (0.26 to 0.30) |
| Active (≥4 days per week) | Inactive (<150 min/week) | 0.04 (0.04 to 0.05) |
| Inactive (<4 days per week) | Active (≥150 min/week) | 0.54 (0.52 to 0.56) |
| Inactive (<4 days per week) | Inactive (<150 min/week) | 0.14 (0.12 to 0.15) |

Supplementary Table 5. The association between adolescent sport and exercise participation and percentage body fat in adulthood, stratified by baseline HUNT survey.

|  | Young HUNT1 | | | |  | Young HUNT3 | | | |
| --- | --- | --- | --- | --- | --- | --- | --- | --- | --- |
| Sport and exercise participation | No. of people | Mean  body fat (%) | Crude  mean  difference | Adjusted^*^ mean difference (95% CI) |  | No. of people | Mean  body fat (%) | Crude  mean  difference | Adjusted^*^ mean difference (95% CI) |
| Days per week |  |  |  |  |  |  |  |  |  |
| <1 day | 422 | 30.2 | 0.0 (ref.) | 0.0 (ref.) |  | 251 | 30.7 | 0.0 (ref.) | 0.0 (ref.) |
| 1 day | 398 | 30.5 | 0.3 | -0.1 (-1.3 to 1.0) |  | 247 | 29.7 | -1.0 | -1.3 (-2.8 to 0.2) |
| 2-3 days | 1076 | 28.9 | -1.3 | -1.5 (-2.4 to -0.6) |  | 714 | 28.1 | -2.7 | -2.7 (-4.0 to -1.5) |
| 4-6 days | 562 | 26.5 | -3.7 | -3.0 (-4.0 to -1.9) |  | 576 | 25.7 | -5.0 | -4.7 (-5.9 to -3.4) |
| Every day | 136 | 25.3 | -4.9 | -3.4 (-5.0 to -1.9) |  | 221 | 22.7 | -8.0 | -5.4 (-7.0 to -3.9) |
| Hours per week |  |  |  |  |  |  |  |  |  |
| <1 hr | 489 | 29.7 | 0.0 (ref.) | 0.0 (ref.) |  | 259 | 31.1 | 0.0 (ref.) | 0.0 (ref.) |
| 1-1.5 hrs | 339 | 30.5 | 0.7 | -0.1 (-1.3 to 1.0) |  | 321 | 29.7 | -1.4 | -1.9 (-3.3 to -0.5) |
| 2-3 hrs | 760 | 29.7 | 0.0 | -0.3 (-1.2 to 0.6) |  | 440 | 27.7 | -3.3 | -3.8 (-5.1 to -2.5) |
| 4-6 hrs | 686 | 27.5 | -2.2 | -2.2 (-3.1 to -1.3) |  | 557 | 36.5 | -4.6 | -4.6 (-5.9 to -4.3) |
| ≥7 hrs | 320 | 25.1 | -4.6 | -3.2 (-4.3 to -2.0) |  | 432 | 24.0 | -7.0 | -5.6 (-6.9 to -4.3 |
| Competing in sports^†^ |  |  |  |  |  |  |  |  |  |
| No | 250 | 31.5 | 0.0 (ref.) | 0.0 (ref.) |  | 351 | 30.0 | 0.0 (ref.) | 0.0 (ref.) |
| No, but used to | 823 | 29.6 | -2.0 | -1.0 (-2.2 to 0.2) |  | 673 | 28.7 | -1.3 | -1.4 (-1.9 to 0.4) |
| Yes | 1156 | 27.1 | -4.4 | -3.0 (-4.1 to -1.9) |  | 950 | 25.4 | -4.6 | -3.5 (-4.6 to -2.5) |

^*^Adjusted for baseline age (continuous), sex (male, female), HUNT survey (Young-HUNT1, Young-HUNT3), and parental education (low, medium, high)

^†^ 400 people with missing data

CI, Confidence interval

Supplementary Table 6. The association between adolescent sport and exercise participation and percentage skeletal muscle mass in adulthood, stratified by baseline HUNT survey.

|  | Young-HUNT1 | | | |  | Young-HUNT3 | | | |
| --- | --- | --- | --- | --- | --- | --- | --- | --- | --- |
| Sport and exercise participation | No. of people | Mean  muscle mass (%) | Crude  mean  difference | Adjusted^*^ mean difference (95% CI) |  | No. of people | Mean  muscle mass (%) | Crude  mean  difference | Adjusted^*^ mean difference (95% CI) |
| Days per week |  |  |  |  |  |  |  |  |  |
| <1 day | 422 | 38.9 | 0.0 (ref.) | 0.0 (ref.) |  | 251 | 38.5 | 0.0 (ref.) | 0.0 (ref.) |
| 1 day | 398 | 38.7 | -0.2 | 0.1 (-0.5 to 0.7) |  | 247 | 39.1 | 0.6 | 0.8 (0.0 to 1.6) |
| 2-3 days | 1076 | 39.6 | 0.7 | 0.9 (0.4 to 1.4) |  | 714 | 40.1 | 1.6 | 1.7 (1.0 to 2.4) |
| 4-6 days | 562 | 41.2 | 2.2 | 1.8 (1.2 to 2.3) |  | 576 | 41.6 | 3.1 | 2.8 (2.1 to 3.5) |
| Every day | 136 | 42.0 | 3.0 | 2.0 (1.1 to 2.9) |  | 221 | 43.5 | 5.0 | 3.3 (2.4 to 4.1) |
| Hours per week |  |  |  |  |  |  |  |  |  |
| <1 hr | 489 | 39.2 | 0.0 (ref.) | 0.0 (ref.) |  | 259 | 38.3 | 0.0 (ref.) | 0.0 (ref.) |
| 1-1.5 hrs | 339 | 38.7 | -0.5 | 0.1 (-0.6 to 0.7) |  | 321 | 39.1 | 0.8 | 1.2 (0.4 to 1.9) |
| 2-3 hrs | 760 | 39.2 | 0.0 | 0.2 (-0.3 to 0.7) |  | 440 | 40.2 | 1.9 | 2.3 (1.5 to 3.0) |
| 4-6 hrs | 686 | 40.5 | 1.2 | 1.3 (0.8 to 1.8) |  | 557 | 41.1 | 2.7 | 2.8 (2.1 to 3.5) |
| ≥7 hrs | 320 | 42.1 | 2.9 | 1.9 (1.3 to 2.5) |  | 432 | 42.6 | 4.3 | 3.4 (2.6 to 4.1) |
| Competing in sports^†^ |  |  |  |  |  |  |  |  |  |
| No | 250 | 38.0 | 0.0 (ref.) | 0.0 (ref.) |  | 351 | 38.9 | 0.0 (ref.) | 0.0 (ref.) |
| No, but used to | 823 | 39.3 | 1.2 | 0.6 (-0.1 to 1.2) |  | 673 | 39.8 | 0.9 | 0.5 (-0.1 to 1.1) |
| Yes | 1156 | 40.8 | 2.7 | 1.8 (1.2 to 2.4) |  | 950 | 41.7 | 2.8 | 2.1 (1.5 to 2.7) |

^*^ Adjusted for baseline age (continuous), sex (male, female), HUNT survey (Young-HUNT1, Young-HUNT3), and parental education (low, medium, high)

^†^ 400 people with missing data

CI, confidence interval

Supplementary Table 7. The association between adolescent sport and exercise participation and body mass index in adulthood, stratified by baseline HUNT survey.

|  | Young-HUNT1 | | | |  | Young-HUNT3 | | | |
| --- | --- | --- | --- | --- | --- | --- | --- | --- | --- |
| Sport and exercise participation | No. of people | Mean  BMI (kg/m^2^) | Crude  mean  difference | Adjusted^*^ mean difference (95% CI) |  | No. of people | Mean  BMI (kg/m^2^) | Crude  mean  difference | Adjusted^*^ mean difference (95% CI) |
| Days per week |  |  |  |  |  |  |  |  |  |
| <1 day | 422 | 27.3 | 0.0 (ref.) | 0.0 (ref.) |  | 251 | 26.7 | 0.0 (ref.) | 0.0 (ref.) |
| 1 day | 398 | 27.2 | -0.1 | 0.1 (-0.6 to 0.7) |  | 247 | 26.3 | -0.4 | -0.3 (-1.2 to 0.6) |
| 2-3 days | 1076 | 26.6 | -0.7 | -0.6 (-1.1 to 0.0) |  | 714 | 25.9 | -0.8 | -0.7 (-1.4 to 0.1) |
| 4-6 days | 562 | 26.3 | -1.1 | -0.9 (-1.5 to -0.3) |  | 576 | 25.4 | -1.3 | -1.1 (-1.9 to -0.4) |
| Every day | 136 | 26.6 | -0.7 | -0.8 (-1.7 to 0.2) |  | 221 | 25.1 | -1.6 | -1.5 (-2.4 to -0.5) |
| Hours per week |  |  |  |  |  |  |  |  |  |
| <1 hr | 489 | 27.2 | 0.0 (ref.) | 0.0 (ref.) |  | 259 | 27.0 | 0.0 (ref.) | 0.0 (ref.) |
| 1-1.5 hrs | 339 | 26.8 | -0.4 | -0.2 (-0.9 to 0.5) |  | 321 | 26.3 | -0.7 | -0.5 (-1.3 to 0.3) |
| 2-3 hrs | 760 | 26.9 | -0.3 | -0.1 (-0.7 to 0.4) |  | 440 | 25.5 | -1.6 | -1.4 (-2.1 to -0.6) |
| 4-6 hrs | 686 | 26.3 | -0.9 | -0.8 (-1.3 to -0.2) |  | 557 | 25.4 | -1.6 | -1.4 (-2.1 to -0.6) |
| ≥7 hrs | 320 | 26.4 | -0.8 | -0.8 (-1.4 to -0.1) |  | 432 | 25.4 | -1.6 | -1.4 (-2.2 to -0.6) |
| Competing in sports^†^ |  |  |  |  |  |  |  |  |  |
| No | 250 | 27.3 | 0.0 (ref.) | 0.0 (ref.) |  | 351 | 26.3 | 0.0 (ref.) | 0.0 (ref.) |
| No, but used to | 823 | 27.1 | -0.2 | -0.3 (-1.0 to 0.4) |  | 673 | 26.2 | -0.1 | -0.1 (-0.8 to 0.5) |
| Yes | 1156 | 26.3 | -1.1 | -1.1 (-1.7 to -0.4) |  | 950 | 25.3 | -1.0 | -0.9 (-1.5 to -0.3) |

^*^Adjusted for baseline age (continuous), sex (male, female), HUNT survey (Young-HUNT1, Young-HUNT3), and parental education (low, medium, high)

^†^ 400 people with missing data

CI, confidence interval; BMI, body mass index

Supplementary Table 8. The association between adolescent sport and exercise participation and body fat, skeletal muscle mass, and body mass index in adulthood for females.

| Sports and exercise participation | No. of people | Mean  body  fat (%) | Crude mean difference | Adjusted^*^ mean difference (95% CI) |  | Mean  muscle mass (%) | Crude mean difference | Adjusted^*^ mean difference (95% CI) |  | Mean  BMI (kg/m^2^) | Crude mean difference | Adjusted^*^ mean difference (95% CI) |
| --- | --- | --- | --- | --- | --- | --- | --- | --- | --- | --- | --- | --- |
| Days per week |  |  |  |  |  |  |  |  |  |  |  |  |
| <1 day | 384 | 35.0 | 0.0 (ref.) | 0.0 (ref.) |  | 35.6 | 0.0 (ref.) | 0.0 (ref.) |  | 26.7 | 0.0 (ref.) | 0.0 (ref.) |
| 1 day | 398 | 34.1 | -0.8 | -0.8 (-1.9 to 0.5) |  | 36.2 | 0.5 | 0.5 (-0.2 to 1.1) |  | 26.6 | -0.2 | -0.1 (-0.8 to 0.6) |
| 2-3 days | 1085 | 32.3 | -2.7 | -2.5 (-3.5 to -1.5) |  | 37.2 | 1.6 | 1.5 (0.9 to 2.0) |  | 25.9 | -0.8 | -0.6 (-1.2 to 0.0) |
| 4-6 days | 626 | 30.8 | -4.2 | -3.8 (-4.9 to -2.6) |  | 38.1 | 2.5 | 2.3 (1.6 to 2.9) |  | 25.4 | -1.3 | -1.0 (-1.6 to -0.3) |
| Every day | 142 | 29.8 | -5.2 | -4.6 (-6.3 to -2.9) |  | 38.6 | 3.0 | 2.7 (1.8 to 3.6) |  | 24.9 | -1.9 | -1.4 (-2.4 to -0.4) |
| Hours per week |  |  |  |  |  |  |  |  |  |  |  |  |
| <1 hr | 412 | 35.2 | 0.0 (ref.) | 0.0 (ref.) |  | 35.6 | 0.0 (ref.) | 0.0 (ref.) |  | 27.0 | 0.0 (ref.) | 0.0 (ref.) |
| 1-1.5 hrs | 425 | 33.7 | -1.5 | -1.2 (-2.4 to 0.0) |  | 36.4 | 0.9 | 0.7 (0.1 to 1.4) |  | 26.2 | -0.8 | -0.6 (-1.3 to 0.1) |
| 2-3 hrs | 731 | 32.7 | -2.4 | -2.2 (-3.2 to -1.1) |  | 37.0 | 1.4 | 1.3 (0.7 to 1.9) |  | 26.0 | -1.0 | -0.8 (-1.4 to -0.2) |
| 4-6 hrs | 726 | 31.1 | -4.0 | -3.7 (-4.8 to -2.7) |  | 37.9 | 2.4 | 2.2 (1.6 to 2.8) |  | 25.5 | -1.2 | -1.3 (-1.9 to -0.7) |
| ≥7 hrs | 341 | 30.1 | -5.1 | -4.5 (-5.7 to -3.2) |  | 38.5 | 3.0 | 2.7 (2.0 to 3.3) |  | 25.3 | -1.7 | -1.3 (-2.0 to -0.5) |
| Competing in sports^†^ | |  |  |  |  |  |  |  |  |  |  |  |
| No | 389 | 34.7 | 0.0 (ref.) | 0.0 (ref.) |  | 35.8 | 0.0 (ref.) | 0.0 (ref.) |  | 26.6 | 0.0 (ref.) | 0.0 (ref.) |
| No, but used to | 876 | 33.3 | -1.4 | -1.3 (-2.4 to -0.3) |  | 36.7 | 0.8 | 0.8 (0.2 to 1.4) |  | 26.3 | -0.4 | -0.5 (-1.1 to 0.2) |
| Yes | 1163 | 30.9 | -3.8 | -3.5 (-4.6 to -2.5) |  | 38.1 | 2.3 | 2.1 (1.6 to 2.7) |  | 25.4 | -1.2 | -1.1 (-1.7 to -0.5) |

^*^ Adjusted for baseline age (continuous), HUNT survey (Young-HUNT1, Young-HUNT3), and parental education (low, medium, high)

^†^ 207 people with missing data

CI, Confidence interval; BMI, body mass index Supplementary Table 9. The association between adolescent sport and exercise participation and body fat, skeletal muscle mass, and body mass index in adulthood for males.

| Sports and exercise participation | No. of people | Mean  body  fat (%) | Crude mean difference | Adjusted^*^ mean difference (95% CI) |  | Mean  muscle mass (%) | Crude mean difference | Adjusted^*^ mean difference (95% CI) |  | Mean  BMI (kg/m^2^) | Crude mean difference | Adjusted^*^ mean difference (95% CI) |
| --- | --- | --- | --- | --- | --- | --- | --- | --- | --- | --- | --- | --- |
| Days per week |  |  |  |  |  |  |  |  |  |  |  |  |
| <1 day | 289 | 24.3 | 0.0 (ref.) | 0.0 (ref.) |  | 42.9 | 0.0 (ref.) | 0.0 (ref.) |  | 27.5 | 0.0 (ref.) | 0.0 (ref.) |
| 1 day | 247 | 23.8 | -0.5 | -0.4 (-1.7 to 0.9) |  | 43.2 | 0.3 | 0.2 (-0.5 to 1.0) |  | 27.4 | -0.1 | -0.1 (-0.8 to 0.7) |
| 2-3 days | 705 | 22.9 | -1.5 | -1.3 (-2.4 to -0.2) |  | 43.8 | 0.9 | 0.7 (0.1 to 1.3) |  | 26.9 | -0.7 | -0.5 (-1.2 to 0.1) |
| 4-6 days | 512 | 20.3 | -4.0 | -3.5 (-4.7 to -2.4) |  | 45.3 | 2.4 | 2.1 (1.5 to 2.7) |  | 26.3 | -1.3 | -1.0 (-1.7 to -0.3) |
| Every day | 215 | 19.7 | -4.7 | -4.0 (-5.3 to -2.6) |  | 45.7 | 2.8 | 2.4 (1.6 to 3.2) |  | 26.2 | -1.3 | -0.9 (-1.7 to -0.1) |
| Hours per week |  |  |  |  |  |  |  |  |  |  |  |  |
| <1 hr | 336 | 24.1 | 0.0 (ref.) | 0.0 (ref.) |  | 43.0 | 0.0 (ref.) | 0.0 (ref.) |  | 27.3 | 0.0 (ref.) | 0.0 (ref.) |
| 1-1.5 hrs | 235 | 23.6 | -0.5 | -0.1 (-1.4 to 1.2) |  | 43.3 | 0.3 | 0.1 (-0.7 to 0.8) |  | 27.3 | 0.0 | 0.2 (-0.6 to 1.0) |
| 2-3 hrs | 469 | 23.1 | -1.0 | -0.8 (-1.9 to 0.3) |  | 43.6 | 0.6 | 0.5 (-0.1 to 1.1) |  | 26.9 | -0.4 | -0.3 (-0.9 to 0.4) |
| 4-6 hrs | 517 | 21.3 | -2.8 | -2.3 (-3.4 to -1.2) |  | 44.7 | 1.7 | 1.4 (0.8 to 2.0) |  | 26.5 | -0.9 | -0.6 (-1.2 to 0.0) |
| ≥7 hrs | 411 | 19.8 | -4.2 | -3.5 (-4.6 to -2.4) |  | 45.6 | 2.6 | 2.1 (1.5 to 2.8) |  | 26.3 | -1.0 | -0.6 (-1.3 to 0.1) |
| Competing in sports^†^ | |  |  |  |  |  |  |  |  |  |  |  |
| No | 212 | 23.2 | 0.0 (ref.) | 0.0 (ref.) |  | 43.6 | 0.0 (ref.) | 0.0 (ref.) |  | 26.9 | 0.0 (ref.) | 0.0 (ref.) |
| No, but used to | 620 | 23.4 | 0.2 | -0.2 (-1.4 to 1.1) |  | 43.5 | -0.1 | 0.1 (-0.6 to 0.8) |  | 27.3 | 0.4 | 0.2 (-0.5 to 0.9) |
| Yes | 943 | 20.8 | -2.4 | -2.7 (-3.8 to -1.5) |  | 45.0 | 1.4 | 1.6 (1.0 to 2.3) |  | 26.3 | -0.6 | -0.7 (-1.4 to 0.0) |

^*^ Adjusted for baseline age (continuous), HUNT survey (Young-HUNT1, Young-HUNT3), and parental education (low, medium, high)

^†^ 193 people with missing data

CI, Confidence interval; BMI, body mass index

Supplementary Table 10. The joint association between adolescent sport and exercise participation and device-measured moderate-to-vigorous physical activity in adulthood with body fat in adulthood, stratified by baseline HUNT survey.

|  | Young HUNT1 | | | |  | Young HUNT3 | | | |
| --- | --- | --- | --- | --- | --- | --- | --- | --- | --- |
| Sport and exercise  participation and MVPA min/week | No. of people | Mean  body fat (%) | Crude  mean  difference | Adjusted^*^ mean difference (95% CI) |  | No. of people | Mean  body fat (%) | Crude  mean  difference | Adjusted^*^ mean difference (95% CI) |
| <1 day per week |  |  |  |  |  |  |  |  |  |
| <150 min | 105 | 35.4 | 0.0 (ref.) | 0.0 (ref.) |  | 58 | 32.4 | 0.0 (ref.) | 0.0 (ref.) |
| 150-299 min | 174 | 29.8 | -5.6 | -4.8 (-6.7 to -2.9) |  | 116 | 28.9 | -3.6 | -3.7 (-6.3 to -1.1) |
| >300 min | 148 | 27.5 | -7.9 | -6.3 (-8.2 to -4.4) |  | 84 | 29.0 | -3.4 | -2.7 (-5.5 to 0.1) |
| 2-3 days per week |  |  |  |  |  |  |  |  |  |
| <150 min | 96 | 33.8 | -1.6 | -1.9 (-4.0 to 0.2) |  | 64 | 32.3 | -0.2 | -0.5 (-3.4 to 2.4) |
| 150-299 min | 249 | 29.6 | -5.8 | -5.0 (-6.8 to -3.2) |  | 134 | 30.1 | -2.4 | -2.8 (-5.3 to -0.2) |
| >300 min | 249 | 25.6 | -9.7 | -8.1 (-9.8 to -6.3) |  | 146 | 25.6 | -6.8 | -5.7 (-8.3 to -3.2) |
| >4 days per week |  |  |  |  |  |  |  |  |  |
| <150 min | 49 | 30.9 | -4.5 | -4.1 (-6.7 to -1.5) |  | 57 | 30.6 | -1.9 | -2.4 (-5.4 to 0.7) |
| 150-299 min | 137 | 27.2 | -8.2 | -6.5 (-8.4 to -4.5) |  | 144 | 26.7 | -5.7 | -4.7 (-7.2 to -2.2) |
| >300 min | 186 | 23.5 | -11.9 | -9.1 (-11.0 to -7.2) |  | 202 | 22.3 | -10.2 | -8.0 (-10.5 to -5.6) |

^*^ Adjusted for baseline age (continuous), sex (male, female), HUNT survey (Young-HUNT1, Young-HUNT3), and parental education (low, medium, high)

CI, Confidence interval; MVPA, moderate-to-vigorous intensity physical activity

Supplementary Table 11. The joint association between adolescent sport and exercise participation and device-measured moderate-to-vigorous physical activity in adulthood with skeletal muscle mass in adulthood, stratified by baseline HUNT survey.

| Sport and exercise  participation and MVPA min/week | Young HUNT1 | | | |  | Young HUNT3 | | | |
| --- | --- | --- | --- | --- | --- | --- | --- | --- | --- |
|  | No. of people | Mean  muscle mass (%) | Crude  mean  difference | Adjusted^*^ mean difference (95% CI) |  | No. of people | Mean  muscle mass (%) | Crude  mean  difference | Adjusted^*^ mean difference (95% CI) |
| <1 day per week |  |  |  |  |  |  |  |  |  |
| <150 min | 105 | 35.8 | 0.0 (ref.) | 0.0 (ref.) |  | 58 | 37.4 | 0.0 (ref.) | 0.0 (ref.) |
| 150-299 min | 174 | 39.0 | 3.2 | 2.7 (1.6 to 3.7) |  | 116 | 39.4 | 2.0 | 2.0 (0.6 to 3.5) |
| >300 min | 148 | 40.5 | 4.6 | 3.5 (2.5 to 4.6) |  | 84 | 39.5 | 2.1 | 1.6 (0.1 to 3.1) |
| 2-3 days per week |  |  |  |  |  |  |  |  |  |
| <150 min | 96 | 36.7 | 0.8 | 1.0 (-0.1 to 2.2) |  | 64 | 37.7 | 0.3 | 0.5 (-1.1 to 2.2) |
| 150-299 min | 249 | 39.1 | 3.3 | 2.8 (1.8 to 3.8) |  | 134 | 38.8 | 1.4 | 1.7 (0.3 to 3.1) |
| >300 min | 249 | 51.5 | 5.7 | 4.5 (3.6 to 5.5) |  | 146 | 41.5 | 4.1 | 3.4 (2.0 to 4.8) |
| >4 days per week |  |  |  |  |  |  |  |  |  |
| <150 min | 49 | 38.3 | 2.5 | 2.2 (0.8 to 3.7) |  | 57 | 38.6 | 1.2 | 1.5 (-0.1 to 3.2) |
| 150-299 min | 137 | 40.8 | 4.9 | 3.7 (2.6 to 4.8) |  | 144 | 40.9 | 3.5 | 2.8 (1.4 to 4.2) |
| >300 min | 186 | 43.0 | 7.2 | 5.2 (4.2 to 6.3) |  | 202 | 43.6 | 6.2 | 4.8 (3.4 to 6.1) |

^*^Adjusted for baseline age (continuous), sex (male, female), HUNT survey (Young-HUNT1, Young-HUNT3), and parental education (low, medium, high)

CI, Confidence interval; MVPA, moderate-to-vigorous intensity physical activity

Supplementary Table 12. The joint association between adolescent sport and exercise participation and device-measured moderate-to-vigorous physical activity in adulthood with body mass index in adulthood, stratified by baseline HUNT survey.

| Sport and exercise  participation and MVPA min/week | Young HUNT1 | | | |  | Young HUNT3 | | | |
| --- | --- | --- | --- | --- | --- | --- | --- | --- | --- |
|  | No. of people | Mean  BMI (kg/m^2^) | Crude  mean  difference | Adjusted^*^ mean difference (95% CI) |  | No. of people | Mean  BMI (kg/m^2^) | Crude  mean  difference | Adjusted^*^ mean difference (95% CI) |
| <1 day per week |  |  |  |  |  |  |  |  |  |
| <150 min | 105 | 28.8 | 0.0 (ref.) | 0.0 (ref.) |  | 58 | 27.1 | 0.0 (ref.) | 0.0 (ref.) |
| 150-299 min | 174 | 26.4 | -2.4 | -2.5 (-3.6 to -1.4) |  | 116 | 24.8 | -2.4 | -2.4 (-3.9 to -0.9) |
| >300 min | 148 | 26.4 | -2.5 | -2.6 (-3.8 to -1.4) |  | 84 | 26.4 | -0.7 | -0.7 (-2.4 to 0.9) |
| 2-3 days per week |  |  |  |  |  |  |  |  |  |
| <150 min | 96 | 27.5 | -1.3 | -1.3 (-2.5 to 0.0) |  | 64 | 27.5 | 0.4 | 0.6 (-1.1 to 2.4) |
| 150-299 min | 249 | 26.5 | -2.4 | -2.3 (-3.4 to -1.3) |  | 134 | 26.1 | -0.9 | -0.8 (-2.3 to 0.7) |
| >300 min | 249 | 25.5 | -3.3 | -3.4 (-4.5 to -2.3) |  | 146 | 24.9 | -2.9 | -2.1 (-3.6 to -0.6) |
| >4 days per week |  |  |  |  |  |  |  |  |  |
| <150 min | 49 | 26.3 | -2.6 | -2.5 (-4.1 to -1.0) |  | 57 | 26.7 | -0.4 | -0.2 (-1.9 to 1.6) |
| 150-299 min | 137 | 26.3 | -2.5 | -2.6 (-3.8 to -1.4) |  | 144 | 25.7 | -1.4 | -1.3 (-2.8 to 0.2) |
| >300 min | 186 | 25.8 | -3.0 | -3.2 (-4.3 to -2.1) |  | 202 | 24.5 | -2.6 | -2.5 (-3.9 to -1.1) |

^*^Adjusted for baseline age (continuous), sex (male, female), HUNT survey (Young-HUNT1, Young-HUNT3), and parental education (low, medium, high)

CI, Confidence interval; BMI, body mass index; MVPA, moderate-to-vigorous intensity physical activity
